# Reasons for Guardian-Relinquishment of Dogs to Shelters: Animal and Regional Predictors in British Columbia, Canada

**DOI:** 10.3389/fvets.2022.857634

**Published:** 2022-04-14

**Authors:** Bailey H. Eagan, Emilia Gordon, Alexandra Protopopova

**Affiliations:** ^1^Animal Welfare Program, Faculty of Land and Food Systems, University of British Columbia, Vancouver, BC, Canada; ^2^The British Columbia Society for the Prevention of Cruelty to Animals, Vancouver, BC, Canada

**Keywords:** animal shelter, dog, human-animal bond, one welfare, relinquishment

## Abstract

Dogs are relinquished to animal shelters for animal-related or guardian-related reasons. Understanding what drives relinquishment patterns is essential for informing intervention opportunities to keep animals with their guardians. Whereas, overall reasons for relinquishment in a given shelter system have been well explored, analysis of human and animal predictors of relinquishing *for a specific reason* has not been previously attempted. We used characteristics of relinquishment including year, population of the relinquishing guardian's region, health status of the dog, breed, age group, weight, and sex to predict reasons for dog relinquishment to British Columbia (BC) Society for the Prevention of Cruelty to Animals (SPCA) shelters across BC between 2008 and 2019 (*n* = 32,081). Relinquishment trends for puppies and adult dogs were also viewed and described. From 2008–2019, the proportion of dogs relinquished relative to total intake remained consistent (range: 31–35%). Primary reasons reported by guardians were having too many dogs (19%), housing issues (17%), personal issues (15%), financial issues (10%), dog behavior (10%), and guardian health (8%). Over years, an increasing proportion of dogs were relinquished for the reason “too many” (OR = 1.16, 95% CI, 1.10–1.23, *p* < 0.001) and “behavior” (OR = 1.34, 95% CI, 1.26–1.43, *p* < 0.001), while a decreasing proportion were relinquished due to financial problems (OR = 0.94, 95% CI, 0.88–1.00, *p* = 0.047). Being a puppy, mixed breed, small, and from a small or medium population center predicted the reason “too many.” Being a senior, Healthy, or from a medium or large population center predicted the reason “housing issues.” Being a non-puppy, Healthy dog in a large population center predicted the reason “personal issues.” Being a puppy, non-Healthy, female, and from a large population center predicted the reason “financial issues.” Being a larger young adult or adult and Healthy predicted the reason “dog behavior.” Being an adult or senior small dog from a small population center predicted the reason “guardian health.” Particularly promising region-specific intervention opportunities include efforts to prevent too many animals in small population centers, improvement of pet-inclusive housing in large population centers, and providing animal care support in large population centers. Accessible veterinary services, including low-cost or subsidized care, likely benefit dog retention across BC.

## Introduction

Understanding why guardians relinquish their pets to shelters remains an active area of scientific and practical inquiry. Shelter intake due to guardian relinquishment in the United States (USA) ranges from 27–50% of total shelter intake (1–3). In 2019 in Canada, approximately 28,000 dogs entered shelters with 35% entering by guardian relinquishment (4). Often relinquishment has been found more to do with human circumstances than a broken bond with the companion animal (5), and guardians reported turning to this option as a last resort (6). Understanding what drives dog relinquishment to shelters is essential in implementing intervention opportunities to keep animals with their guardians.

Previous research on the primary reasons reported by guardians for the relinquishment of dogs generally falls into two categories: guardian-related or animal-related (7). Housing-related issues are often reported to be a primary reason for the relinquishment of dogs and include moving, landlord or building regulation issues, and an inability to secure pet-friendly housing (8, 9). Securing pet-friendly housing is problematic in many communities, likely because of the worry of increased damages by tenants (10). However, allowing pets in rental units has been demonstrated to result in a lower turnover of tenants, increase the size of the tenant market, and has been suggested to result in increased revenue for landlords as pet-friendly housing is often more expensive than pet-prohibited housing. Consequently, pet-owning renters often report struggling to find suitable places to live and end up compromising on quality, location, and safety (11, 12), or even relinquishing their companion animal to the shelter.

Personal issues such as not enough time or too much unwanted responsibility for animal care, life changes such as a new baby, divorce, or legal challenges may result in dog relinquishment to shelters. The lack of time for the dog and owner personal issues (1, 13) are frequently reported among the top reasons for dog relinquishment to shelters. Other issues, such as the cost of care for dogs, are commonly reported reasons for relinquishment (1, 5) and have been cited as reasons guardians do not access routine veterinary care (14). Further, the guardian's health such as allergies, injuries, illness or death may result in a dog's relinquishment to a shelter. However, in a systematic review and meta-analysis, guardian health/illness had a low overall estimate for causing relinquishment to shelters (15).

A commonly reported reason for dog relinquishment is the guardian having too many animals (hereinafter called “too many”) (15, 16). This reason indicates a guardian having more animals than they can care for, or a litter of puppies they are not able to keep. While all reasons a guardian may relinquish for having too many dogs is unknown, it is likely that relinquishments for the reason of too many provide evidence of overpopulation in a region. Humane Canada reports that most of Canada does not have dog overpopulation, and in some cases, there is a shortage of dogs available for adoption (16). However, it is unknown if this varies across regions.

Behavioral problems have been reported to be the cause for the relinquishment of dogs to shelters (1, 5, 17, 18), and relinquishment may, in some cases, be due to only one behavioral issue (18). Common guardian-reported behavioral reasons for the relinquishment of dogs include inappropriate elimination (19, 20), unwanted chewing (17, 20), aggressive behavior (1, 20), separation anxiety (20) and fearfulness (21). Guardians relinquishing their animals to shelters report not having the time or money to fix behavioral problems (6). However, professional training and behavior consulting result in fewer behavioral problems (22). Despite this, a minority of people seek professional help (23). In a study conducted in BC on the general population of dog guardians, only 39% received help from a professional when training their dog (24).

Other animal-related reasons for the relinquishment of dogs to shelters have been found to include issues with the animal's health alone (unrelated to cost, but due to the issue itself) or specific characteristics such as being reproductively intact (1, 19). Reasons for dog relinquishment are often complicated and likely include a combination of more than one of the drivers mentioned above. Other factors known to contribute to guardian relinquishment of dogs include human demographics (13), duration of guardianship, expectations, household characteristics and care (19), a weakened human-animal bond (17), or social vulnerability of the guardian (25).

Whereas, overall reasons for relinquishment and their proportions in a given shelter system or a community have been well explored, analysis of data on human and animal predictors of relinquishing *for a specific reason* has not been previously attempted. Investigating the predictors of each particular reason for dog relinquishment may help uncover the likely complicated dynamics contributing to these intakes of dogs in BC.

While a detailed understanding of the factors contributing to relinquishment is needed to inform intervention strategies, little is known about reasons for the relinquishment of dogs in BC, Canada, including how animal characteristics such as size, age, suspected breed, sex, and health status are related to relinquishment patterns. Additionally, BC is a large Canadian province with a wide range of population centers, and likely, regional trends across the province further contribute to dog relinquishment. Country has been found to be a significant source of variation for the reasons for dog relinquishment (15). In a scoping review of published research on companion animal relinquishment, Canada, South America, and Asia were identified as regions lacking studies on the reasons for the relinquishment of cats and dogs to shelters (8). A limited number of studies relating to guardian relinquishment in Canada suggests a need for prioritizing research in this area due to the likely potential for geographical differences in companion animal relinquishment patterns (8), and potential relationships between dog characteristics and relinquishment reason.

In Canada, a national survey conducted in 2016 collected primary reasons reported by guardians for guardian relinquishment of cats and dogs to shelters, which provided a general count for relinquishment categories summarizing broad themes (9). The primary reasons for the relinquishment of dogs and cats to shelters in Canada were housing issues, companion animal took too much time or responsibility, financial issues, undesirable animal behavior, poor owner health, among others (9). However, a more detailed understanding of BC, specifically, would allow for regional comparisons. British Columbia (BC), Canada, has some of the lowest vacancy rates (26), the highest rent and purchasing costs in Canada (27), and a lack of pet-friendly housing options (28). Currently, the provincial Residential Tenancy Act (29) allows for restrictions of the size, type, and the number of pets in a rental property across BC. While the extent of restrictions has not been quantified in BC, in the USA, even among pet-friendly housing, approximately 50% are restricted by breed and size. These issues may be further exacerbated if the proportion of renters increases, a likely scenario in areas such as BC where the number of multi-unit registered homes is increasing (30).

Ly et al. (25) found that individual communities may be predisposed to guardian-related risks for relinquishment due to existing vulnerabilities, and relinquishment has been demonstrated to differ by the community (5, 31). Therefore, the interventions and community need likely look different depending on the specific relinquishment reason on a practical level. Demographic differences affect the use of animal shelter services by animal guardians, including guardian-related reasons for relinquishment such as housing issues (25). Further analysis on regional-specific factors relating to relinquishment is needed.

Our study aimed to investigate the specific possible risk factors, including dog age, spay or neuter status, health status, dog size, sex, suspected breed and population center size that predict guardian relinquishment for particular reasons across BC, Canada. We hypothesized that multiple unique characteristics will predict each primary reason for guardian relinquishment and that regional differences in reasons will be evident.

## Methods

As this project included analysis of data excluding human identifiers, this project was deemed exempt from the University of British Columbia Behavioral Research Ethics Board review. Permission for using the ShelterBuddy database (32) was received from the British Columbia Society for the Prevention of Cruelty to Animals (BC SPCA). The BC SPCA is an animal shelter organization in BC that includes 34 animal shelter locations and two foster-based locations. Historical data were gathered and guardian relinquishment records between 2008 and 2019 were extracted from the ShelterBuddy database. Data extracted included dog ID, name, received date, incoming region, relinquishment reason, Asilomar Accords category (33, 34), breed, age group, weight, sex, and incoming spay/neuter status. Asilomar Accords category was used as a measure of health status, and was assigned on intake by shelter staff. Asilomar Accords categories include Healthy (H) when a dog is demonstrating no behavioral or health issues when entering the shelter, Treatable-Rehabilitatable (TR) if a dog is not currently healthy but likely to become so with treatment (e.g., abscessed tooth), Treatable-Manageable (TM) if they are not healthy, not likely to become so, but management is possible (e.g., Cushing's Disease), or Unhealthy-Untreatable (UU) if they have behavioral or medical issues that indicates they are unlikely or unable to recover in shelter (e.g., metastatic cancer) (33, 34). No personal information relating to the relinquishing guardian was extracted or viewed.

Dogs were included as guardian-relinquished if they were brought to the shelter by their guardian, relinquished to an animal care officer by their guardian to be brought to the shelter, or relinquished by their guardian in the field. As the BC SPCA organization has included abandonment and relinquishment for euthanasia in their organizational guardian relinquishment classification, for consistency with published definitions (8), this was included as relinquishment.

Relinquishment trends over time between 2008 and 2019 were viewed and described to observe total intake and the proportion of annual intake due to guardian relinquishment over time. For descriptive purposes, to illustrate differences in relinquishment trends for puppies compared to adult dogs (including young adult, adult and senior), overall proportions of reasons for the relinquishment for puppies and adult dogs were viewed and described separately.

The data were viewed descriptively, separated by reasons for relinquishment. Relationships between predictor shelter variables age group (puppy, young adult, adult and senior), Asilomar Accords categories (H, TR, TM or UU), suspected breed (mixed or purebred), size of population center of relinquishment (small, medium or large), sex (male or female), size (smaller or larger) and year were assessed through binary logistic regression analyses for the primary reasons for relinquishment (“too many,” “housing issues,” “personal issues,” “financial issues,” “dog behavior,” and “guardian health”; total of six regression models). The estimated age group was classified as “puppy” if recorded on intake as “puppy” or “baby” (approximately under 6 months), “young adult” if recorded on intake as “young adult” or “juvenile” (~6 months to 3 years), “adult” if recorded as so (~3–8 years), and “senior” if recorded on intake as “senior” or “geriatric” (approximately over 8 years).

Dog weights were visually assessed and were strongly bimodal, separating at 20 kg. Therefore, 20 kg were considered “smaller” dogs, and dogs over 20 kg were deemed “larger” dogs. Size of the population center was included to assess if characteristics of the area was a factor relating to dog relinquishment. Population size of the region was classified by searching the region of the shelter in Statistics Canada (35). The population was separated into three groups based on the size of the population of the region including “small” if between 1,000 and 29,000, “medium” if between 30,000 and 99,000, and “large urban” (hereinafter called “large”) if 1,00,000 people or more (36).

All statistical analysis was conducted in R Studio (Version 1.4.1106). Binary logistic regression assessed relationships between predictor variables and primary reasons for relinquishment. Relationships were considered statistically significant at *p* < 0.05. Risk ratios and 95% confidence intervals were calculated, and plotted (**Figures 4**–**9**). As spay/neuter status and age group variables were closely linked (with puppies making up the majority of intact animals entering the shelter), spay/neuter statuses were not included as a predictor variable.

## Results

**Figure 1:**
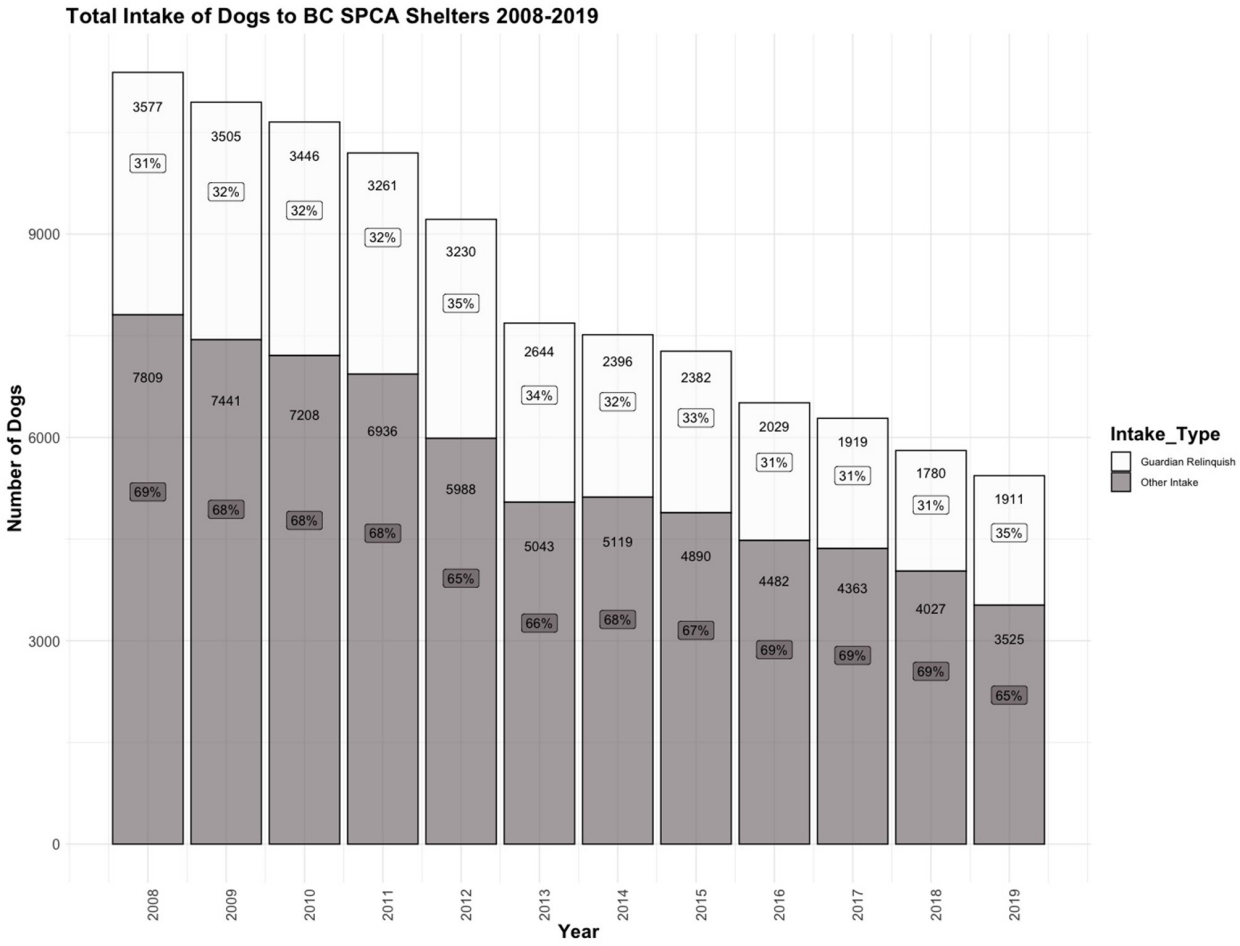
The number of dogs admitted to the BC SPCA between 2008 and 2019. The number of dogs known to be relinquished due to guardian relinquishment is represented in white, and the number of dogs entering the shelter by other intake sources is represented in gray. The number of dogs for each intake type by year is labeled on the corresponding bars in black text, and the percentage (%) of total intake of each intake type are labeled on the corresponding bars in boxes.

Between 2008 and 2019, the total annual intake of dogs to BC SPCA shelters has steadily decreased. However, the proportion of that intake due to guardian relinquishment has remained primarily consistent (Figure 1). Of the total dogs relinquished, the proportion relinquished between 2008 and 2019 has remained between 31 and 35%, with the highest reported proportion due to guardian relinquishment in 2012 and 2019.

In total, 98,911 dogs entered 36 BC SPCA shelters between 2008 and 2019. Of that, 32,081 dogs were relinquished by guardians, 28,304 (88%) of which included a primary reason for relinquishment entered by the BC SPCA staff. In consultation with BC SPCA staff, relinquishment reasons initially provided upon relinquishment from the guardian were classified into a guardian-related reason (*n* = 23,392, 73%), if primarily due to human factors, or an animal-related reason (*n* = 3,433, 11%), if primarily due to animal factors. Further, reasons were sorted into categories and sub-categories of the primary reason for relinquishment. Dogs were relinquished for the primary reason of 1) “too many” (*n* = 6,179, 19%) 2) “housing issues” (*n* = 5,309, 17%), 3) “personal issues” (*n* = 4,955, 15%), 4) “can't afford” (*n* = 3,311, 10%), 5) “behavior” (*n* = 3,213, 10%), 6) “guardian health” (*n* = 2,449, 8%), 7) “no longer wanted” (*n* = 1,189, 4%), 8) “animal health” (*n* = 148, 0.5%), 9) “animal characteristics” (*n* = 72, 0.2%), 10) “community dog” (*n* = 5, 0.01%), or “other” (*n* = 3,777, 12%).

When dogs entered the BC SPCA, staff entered data into the ShelterBuddy database, including estimated age group, Asilomar Accord category, weight, and suspected breed. Classifications of dog characteristics are summarized in Table 1. In some cases, data for one or more dog characteristics were not available or not entered by BC SPCA staff on intake. Therefore, not all categories of dog characteristics total *n* = 32,081 (100%).

**Table 1:** Classification of dog characteristics and their percentage within each category and within the total population (*n* = 32,081).

**Category**	**Characteristic**	***n***	**Within category %**	**Within guardian relinquished population %(*n =* 32,081)**
Age group	Puppy	8,485	26.7	26.4
	Young adult	7,622	24.0	23.7
	Adult	12,189	38.4	37.9
	Senior	3394	10.7	10.5
Spay/neuter	Yes	21,226	67.0	66.1
status	No	10,443	32.9	32.5
Asilomar	Healthy	2,918	38.0	9.0
accord category	Treatable-rehabilitatable	3,321	43.2	10.3
	Treatable-manageable	1,101	14.3	3.4
	Unhealthy-untreatable	331	4.3	1.0
Dog size	Smaller	7,785	58.0	24.2
	Larger	5,627	41.9	17.5
Dog sex	Female	15,480	48.4	48.2
	Male	16,473	51.5	51.3
Breed	Suspected purebred	1,048	3.2	3.2
	Mixed	31,033	96.7	96.7
Population	Small	13,539	42.2	42.2
center size	Medium	9,846	30.7	30.6
	Large	8,678	27.0	27.0

Of all dogs relinquished in small population centers, 8,192 (61%) were previously spayed or neutered compared to *n* = 6,569 (67%) in medium population centers, and *n* = 6,461 (74%) in high population centers. Of dogs over approximately 6 months old (age group of young adult, adult or senior) relinquished in small population centers, 6,373 (70%) were previously spayed or neutered compared to 5,204 (74%) in medium population centers, and 5,462 (80%) in high population centers.

**Figure 2:**
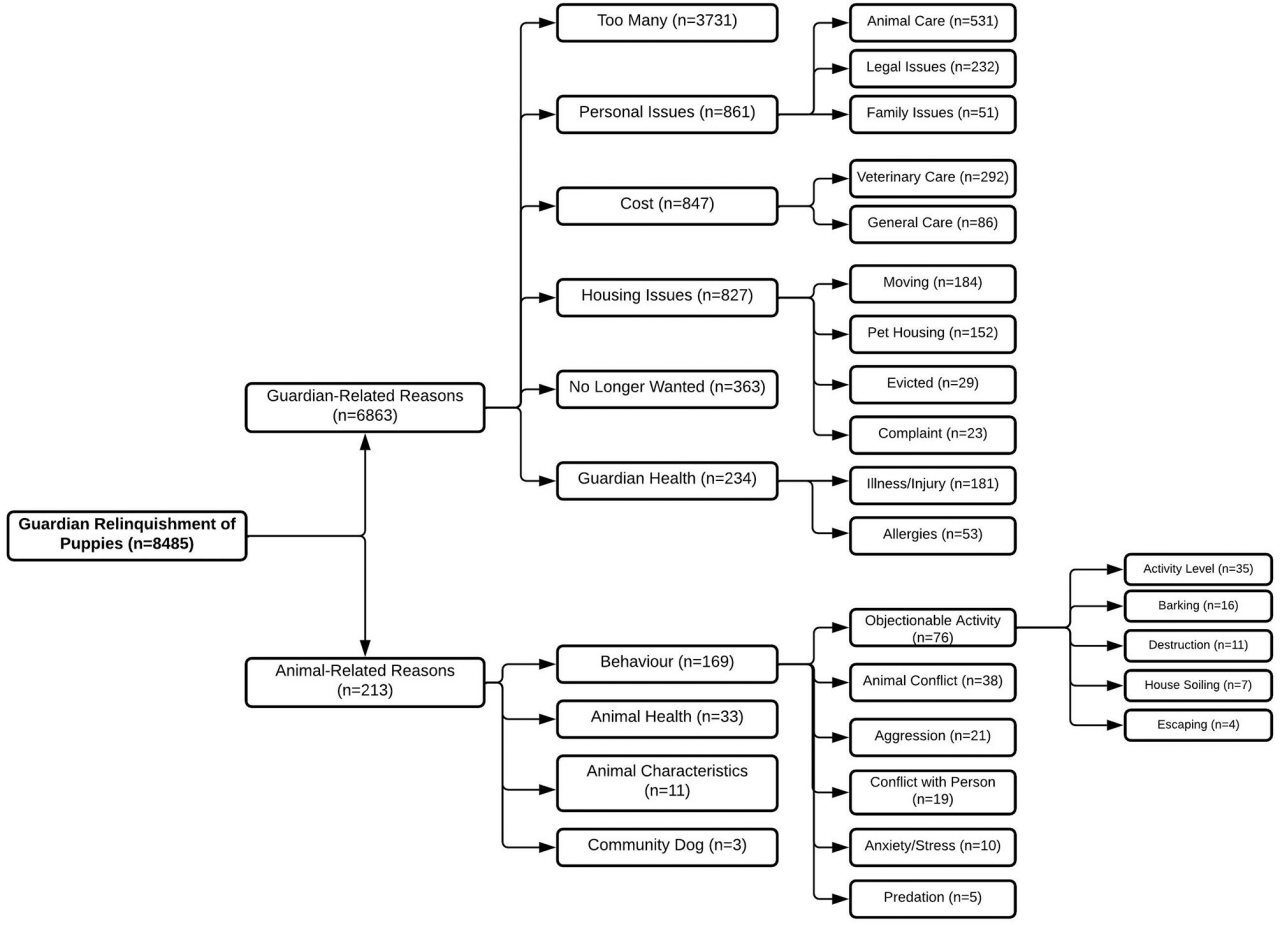
The number of puppies relinquished to the BC SPCA between 2008 and 2019 (*n* = 8,485). Relinquishment reasons include reasons relating to the guardian (*n* = 6,863) and reasons relating to the animal (*n* = 213). Where enough information was provided for further classification, the number of dogs and puppies relinquished for sub-reasons is included.

**Figure 3:**
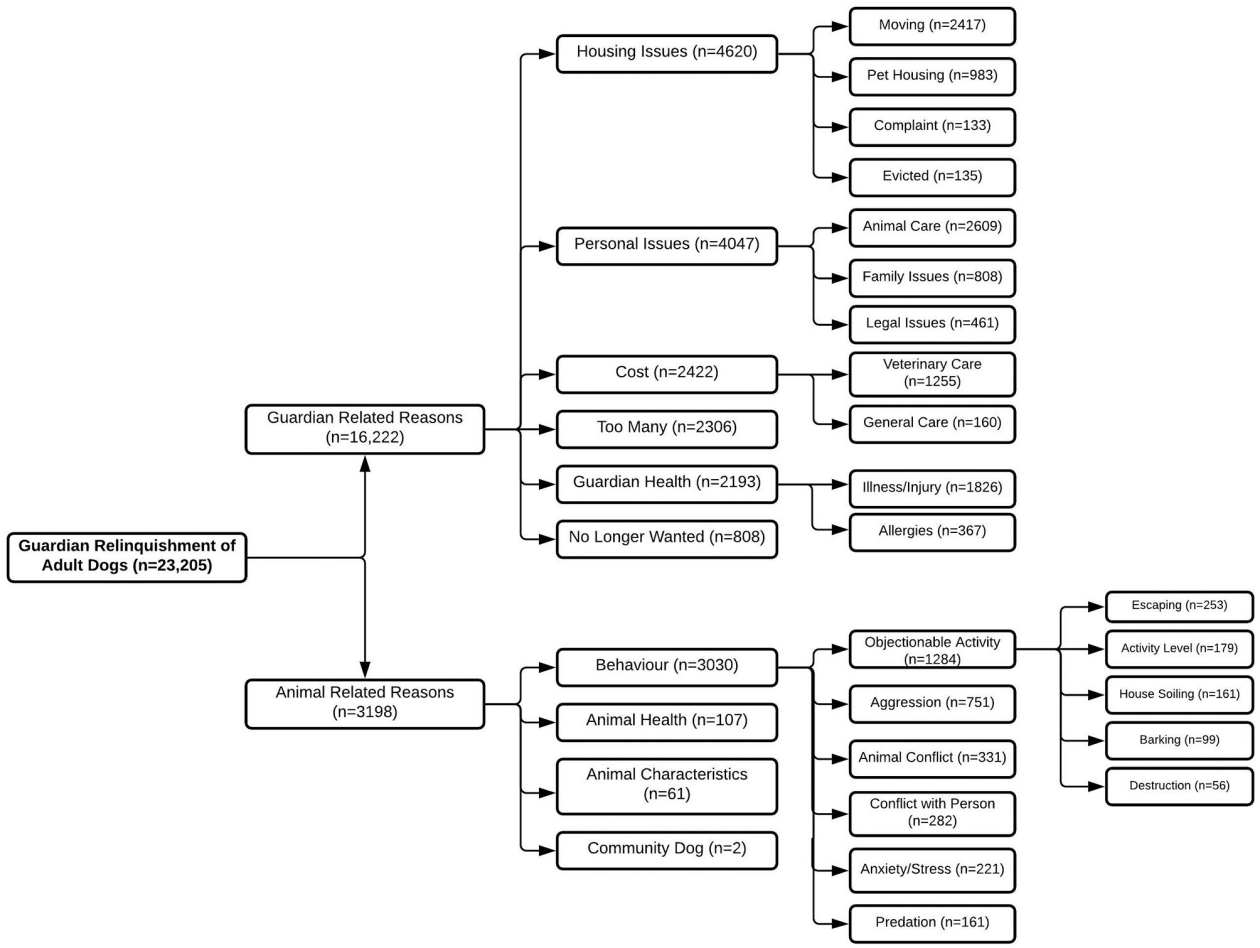
The number of adult dogs relinquished to the BC SPCA between 2008 and 2019 (*n* = 23,205). Relinquishment reasons include reasons relating to the guardian (*n* = 16,222) and reasons relating to the animal (*n* = 3,198). Where enough information was provided for further classification, the number of dogs relinquished for sub-reasons is included.

For both adult dogs and puppies, the reasons for relinquishment were predominately guardian-related (Figures 2, 3). However, the guardian-related reason of too many made up the majority of guardian relinquishments of puppies, while housing issues made up the majority of reasons for adult dogs. Personal issues were the second most common guardian-related reason for relinquishment for both adult dogs and puppies. Of animal-related reasons, behavior was the primary reason for relinquishment of both adult dogs and puppies, with objectionable activities such as activity level, barking, destruction, and escaping being the primary issues for both adult dogs and puppies.

**Figure 4:**
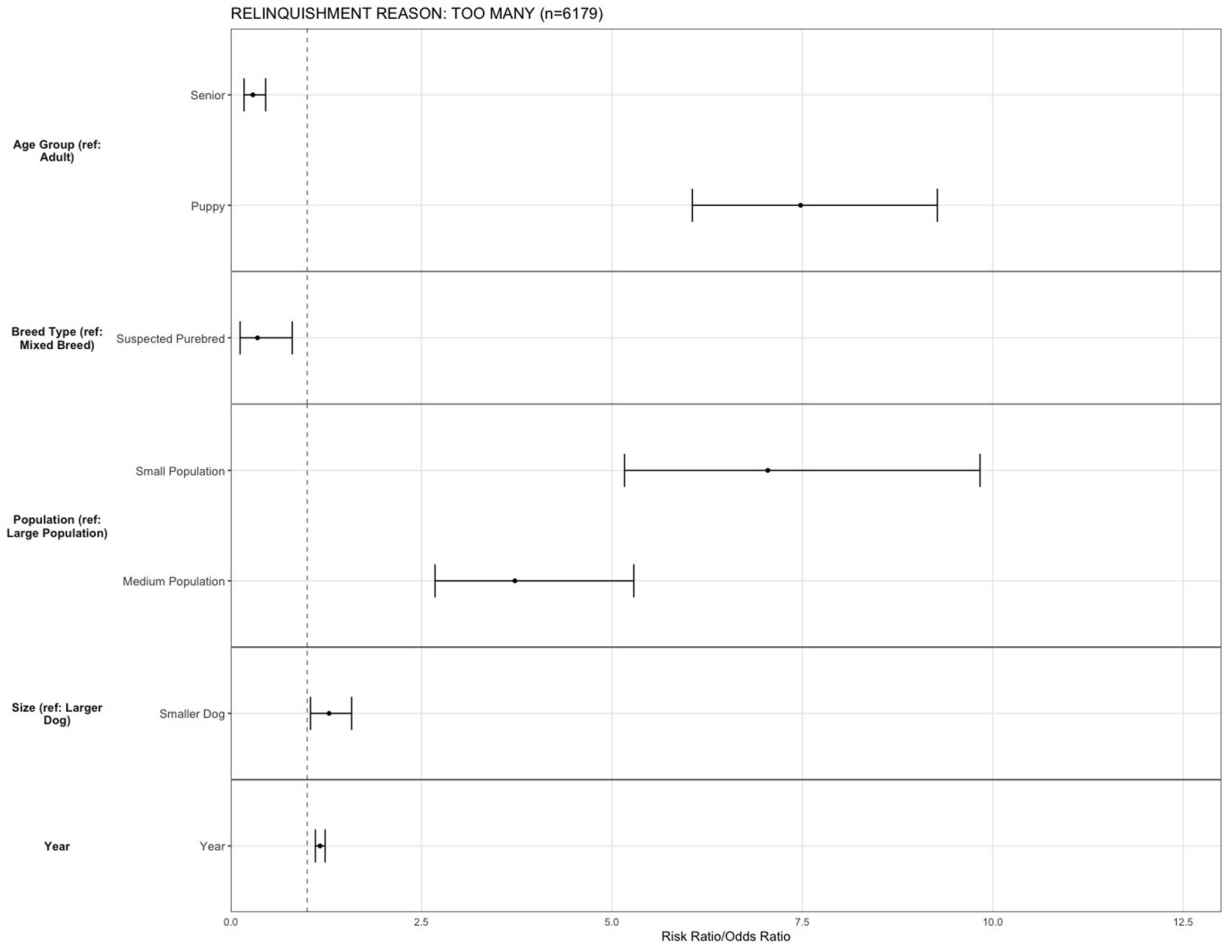
The association between relinquishment reason and characteristics for puppy and adult dogs relinquished by guardians for the reason of “too many” (*n* = 6,179). Characteristics include age group, Asilomar Accords category, breed type, dog size, sex, population center size, and year. The results of the logistic regression model are shown. Data are presented by risk ratios and their 95% confidence interval (bars); *p* < 0.05 when 95% confidence interval does not cross the vertical dotted line. A risk ratio >1 suggests an increased risk of relinquishment of that the characteristic category, while a risk ratio <1 suggests a reduced risk. Non-statistically significant results are excluded from the plot.

**Figure 5:**
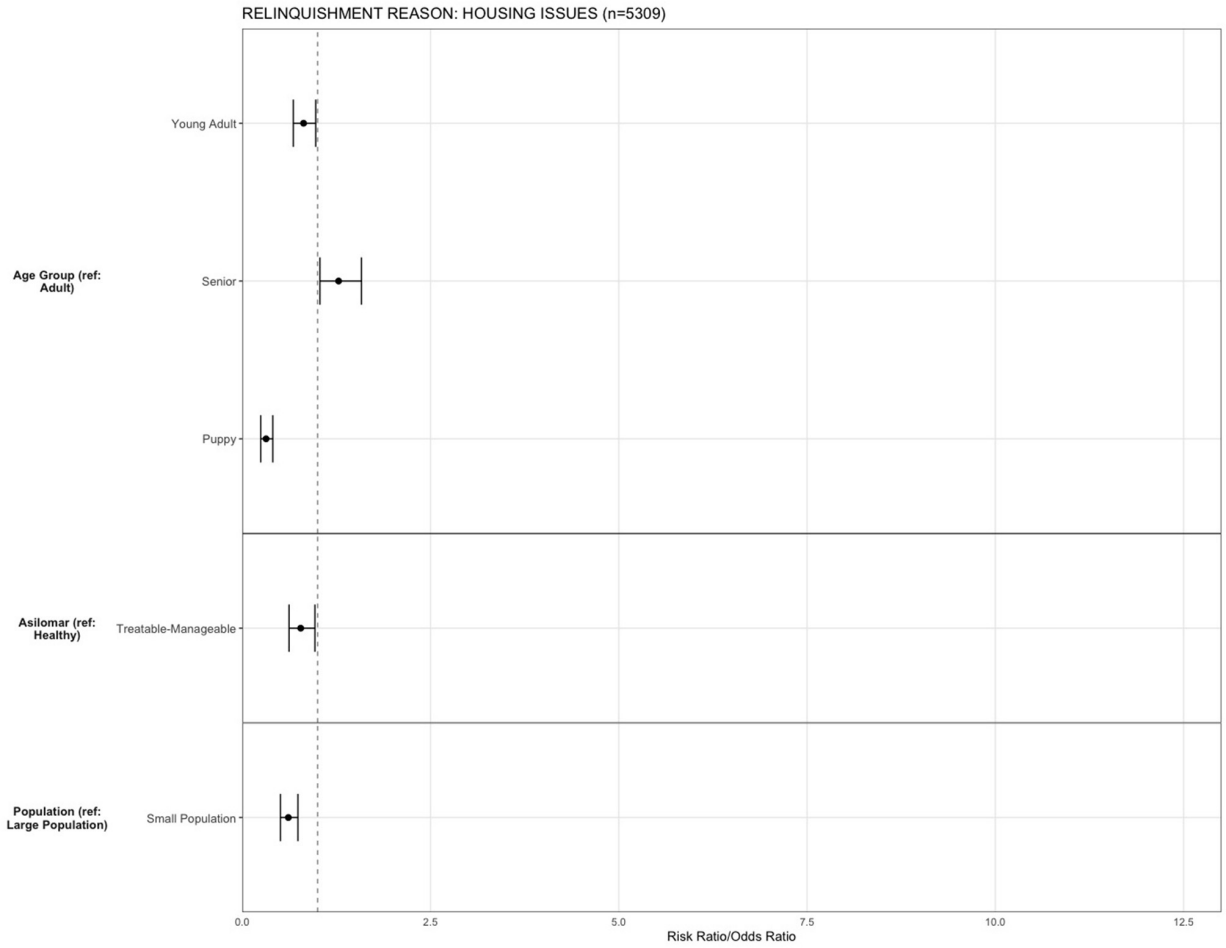
The association between relinquishment reason and animal characteristics for puppies and dogs relinquished for the reason of “housing issues” (*n* = 5,309). Characteristics include age group, Asilomar Accords category, breed type, dog size, sex, population center size, and year. The results of the logistic regression model are shown. Data are presented by risk ratios and their 95% confidence interval (bars); *p* < 0.05 when 95% confidence interval does not cross the vertical dotted line. A risk ratio >1 suggests an increased risk of relinquishment of that the characteristic category, while a risk ratio <1 suggests a reduced risk. Non-statistically significant results are excluded from the plot.

**Figure 6:**
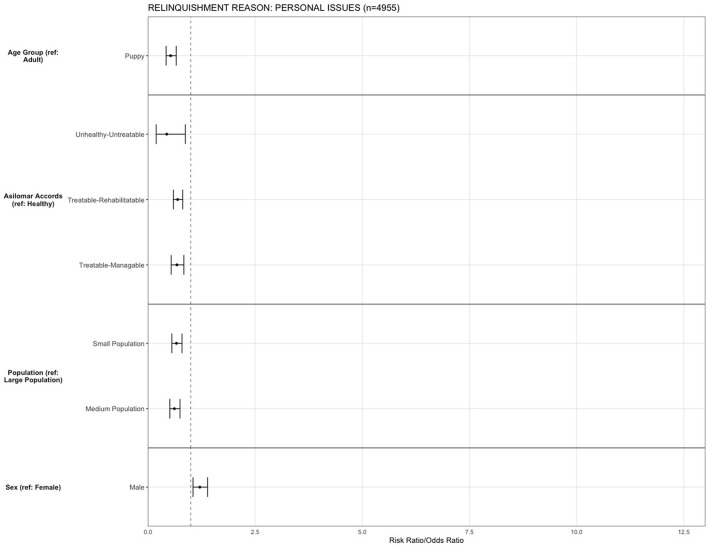
The association between relinquishment reason and animal characteristics for puppies and dogs relinquished for the reason of “personal issues” (*n* = 4,955). Characteristics include age group, Asilomar Accords category, breed type, dog size, sex, population center size, and year. The results of the logistic regression model are shown. Data are presented by risk ratios and their 95% confidence interval (bars); *p* < 0.05 when 95% confidence interval does not cross the vertical dotted line. A risk ratio >1 suggests an increased risk of relinquishment of that the characteristic category, while a risk ratio <1 suggests a reduced risk. Non-statistically significant results are excluded from the plot.

**Figure 7:**
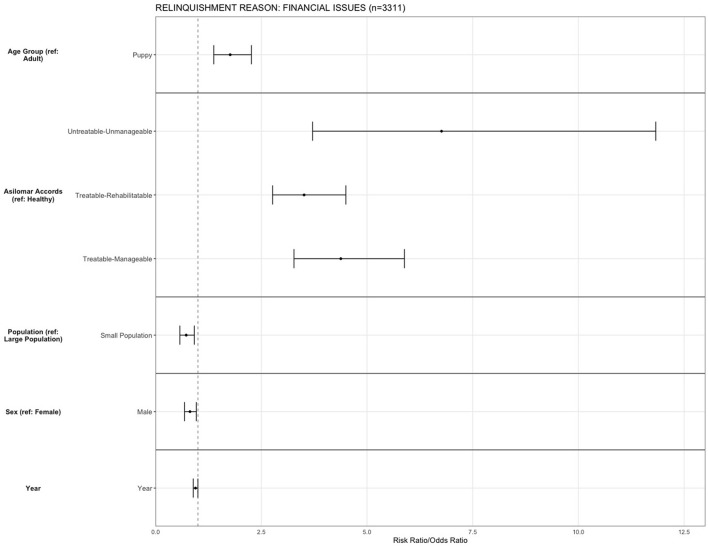
The association between relinquishment reason and animal characteristics for puppies and dogs relinquished for the reason of “financial issues” (*n* = 3,311). Characteristics include age group, Asilomar Accords category, breed type, dog size, sex, population center size, and year. The results of the logistic regression model are shown. Data are presented by risk ratios and their 95% confidence interval (bars); *p* < 0.05 when 95% confidence interval does not cross the vertical dotted line. A risk ratio >1 suggests an increased risk of relinquishment of that the characteristic category, while a risk ratio <1 suggests a reduced risk. Non-statistically significant results are excluded from the plot.

**Figure 8:**
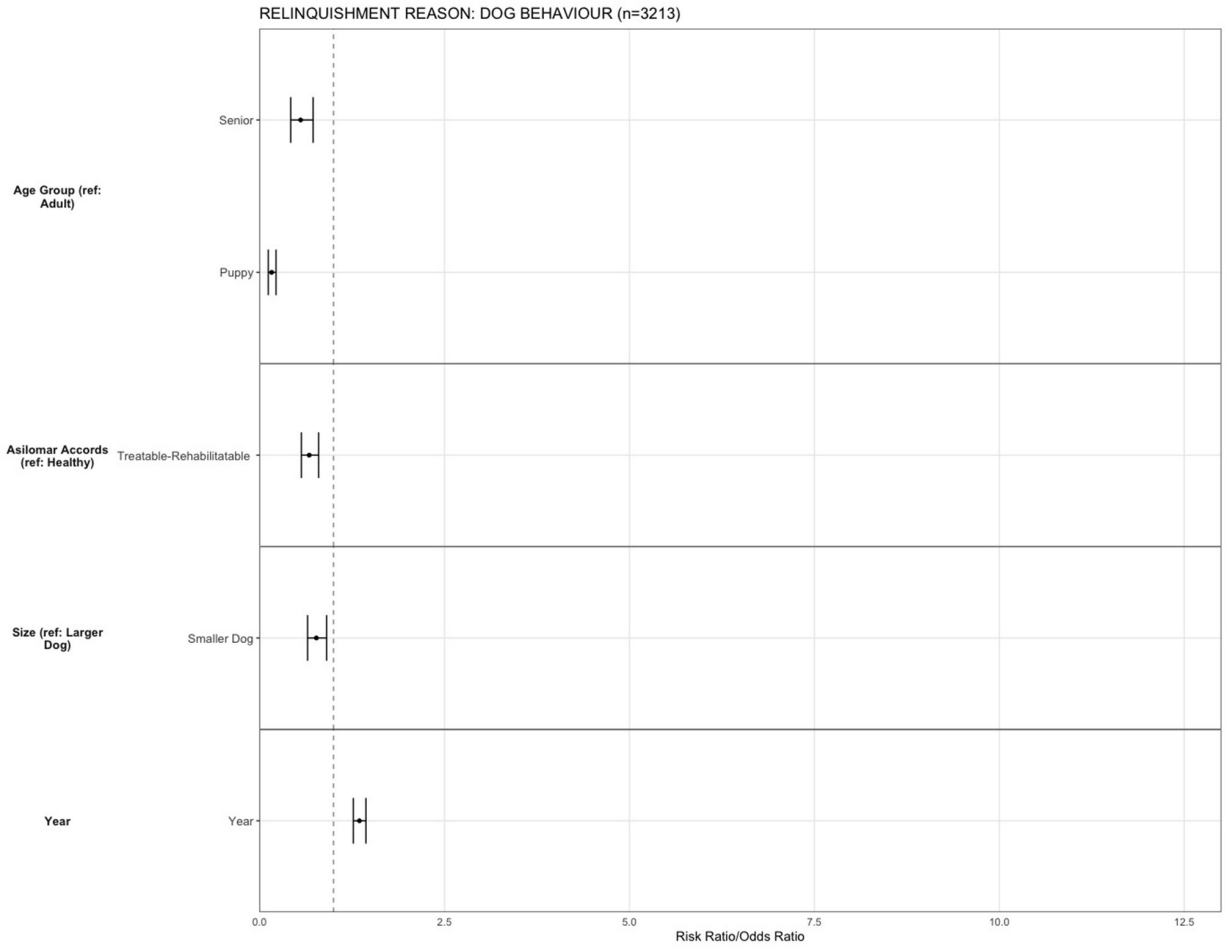
The association between relinquishment reason and animal characteristics for puppies and dogs relinquished for the reason of “dog behavior” (*n* = 3,213). Characteristics include age group, Asilomar Accords category, breed type, dog size, sex, population center size, and year. The results of the logistic regression model are shown. Data are presented by risk ratios and their 95% confidence interval (bars); *p* < 0.05 when 95% confidence interval does not cross the vertical dotted line. A risk ratio >1 suggests an increased risk of relinquishment of that the characteristic category, while a risk ratio <1 suggests a reduced risk. Non-statistically significant results are excluded from the plot.

**Figure 9:**
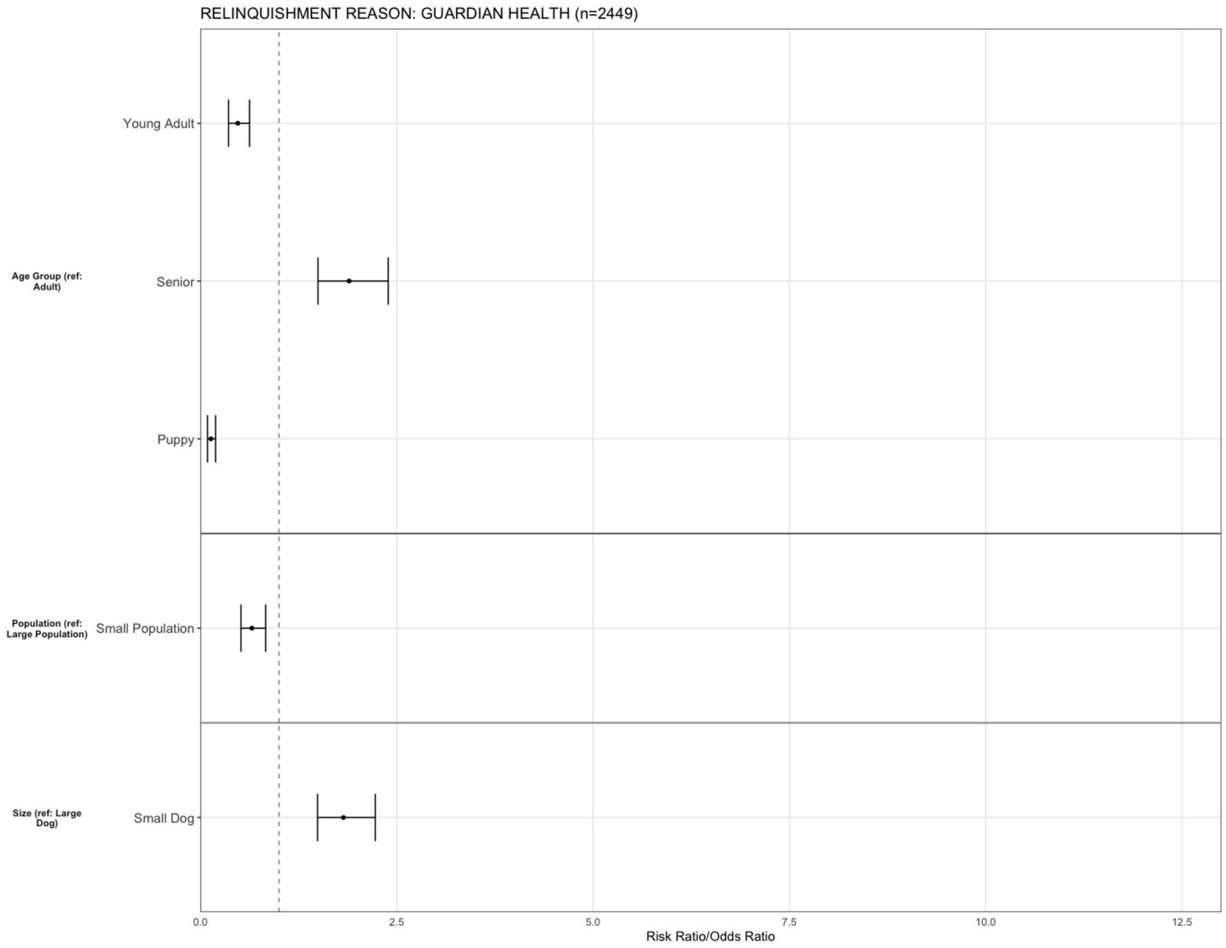
The association between relinquishment reason and animal characteristics for puppies and dogs relinquished for the reason of “guardian health” (*n* = 2,449). Characteristics include age group, Asilomar Accords category, breed type, dog size, sex, population center size, and year. The results of the logistic regression model are shown. Data are presented by risk ratios and their 95% confidence interval (bars); *p* < 0.05 when 95% confidence interval does not cross the vertical dotted line. A risk ratio >1 suggests an increased risk of relinquishment of that the characteristic category, while a risk ratio <1 suggests a reduced risk. Non-statistically significant results are excluded from the plot.

The logistic regression models' results assess the relationship between the primary reasons for relinquishment (for guardian relinquishment reasons accounting for >2,000 dogs between 2008 and 2019) and predictor variables age group, Asilomar Accords category, suspected breed, sex, dog size, and population size of relinquishment region are represented in Figures 4–**9**.

For the reason “too many,” being a puppy (OR = 7.47, 95% CI, 6.05–9.27, *p* < 0.001) increased the risk of relinquishing an animal, and being a senior dog (OR = 0.286, 95% CI, 0.17–0.454, *p* < 0.001) decreased the risk, compared to adult dogs. Smaller dogs (OR = 1.28, 95% CI, 1.04–1.58, *p* = 0.017) were more likely to be relinquished compared to larger dogs, and suspected purebred dogs (OR = 0.347, 95% CI, 0.11–0.80, *p* = 0.027) were less likely to be relinquished for this reason than suspected mixed breeds. The risk for relinquishment was increased in small population (OR = 7.04, 95% CI, 5.16–9.83, *p* < 0.001) and medium population centers (OR = 3.72, 95% CI, 2.67–5.28, *p* < 0.001) compared to large population centers. There was an increase in relinquishment for the reason of “too many” over the years (OR = 1.16, 95% CI, 1.10–1.23, *p* < 0.001) (Figure 4).

For the reason of “housing issues,” being a young adult (OR = 0.81, 95% CI, 0.67–0.97, *p* = 0.026) and puppy (OR = 0.31, 95% CI, 0.24–0.40, *p* < 0.001) decreased the risk of relinquishing an animal, and being a senior (OR = 1.27, 95% CI, 1.03–1.58, *p* = 0.024) increased the risk compared to adult dogs. The risk for relinquishment due to “housing issues” was decreased in small population centers (OR = 0.61, 95% CI, 0.50–0.73, *p* < 0.001) compared to large population centers, and TM dogs were less likely to be relinquished for the reason of housing issues compared to H (OR = 0.77, 95% CI, 0.62–0.96, *p* = 0.023) (Figure 5).

For the reason of “personal issues,” being a puppy (OR = 0.52, 95% CI, 0.42–0.65, *p* < 0.001) decreased the risk of relinquishing an animal compared to adult dogs, and being a male dog (OR = 1.2, 95% CI, 1.05–1.38, *p* = 0.007) increased the risk of being relinquished for personal issues compared to being a female. Compared to H, dogs that were TR (OR = 0.69, 95% CI, 0.59–0.81, *p* < 0.001), TM (OR = 0.67, 95% CI, 0.54–0.83, *p* < 0.001), and UU (OR = 0.43, 95% CI, 0.19–0.87, *p* = 0.030) were less likely to be relinquished. The risk for relinquishment for “personal issues” was decreased in small (OR = 0.66, 95% CI, 0.55–0.79, *p* < 0.001) and medium population centers (OR = 0.61, 95% CI 1.05–1.38, *p* < 0.001) compared to large population centers (Figure 6).

For the reason of “financial issues,” being a puppy (OR = 1.76, 95% CI, 1.37–2.26, *p* ≤ 0.001) increased the risk of relinquishing an animal compared to adult dogs. Compared to H, dogs that were TR (OR = 3.5, 95% CI, 2.76–4.49, *p* < 0.001), TM (OR = 4.38, 95% CI, 3.27–5.88, *p* < 0.001), and UU (OR = 6.76, 95% CI, 3.71–11.82, *p* < 0.001) were more likely to be relinquished. The risk for relinquishment was decreased in small population centers (OR = 0.72, 95% CI, 0.68–0.916, *p* = 0.006) compared to large population centers, and male dogs (OR = 0.81, 95% CI, 0.72–0.99, *p* = 0.016) were less likely to be relinquished for financial reasons compared to females (Figure 7). Over years, there was a slight decrease in the likelihood of relinquishment for financial reasons (OR = 0.94, 95% CI, 0.88–1.00, *p* = 0.047).

For the reason of “dog behavior,” being a puppy (OR = 0.16, 95% CI, 0.01–0.22, *p* < 0.001) and senior (OR = 0.55, 95% CI, 0.42–0.72, *p* < 0.001) decreased the risk of relinquishing an animal compared to adult dogs. Compared to H, dogs that were TR (OR = 0.67, 95% CI, 0.56–0.78, *p* < 0.001) were less likely to be relinquished for dog behavior issues. Smaller dogs (OR = 0.76, 95% CI, 0.64–0.90, *p* = 0.001) were less likely to be relinquished for the reason of “dog behavior” compared to large dogs. There was an increase in relinquishment for the reason of “dog behavior” over the years (OR = 1.34, 95% CI, 1.26–1.43, *p* < 0.001) (Figure 8).

For the reason of “guardian health,” being a puppy (OR = 0.13, 95% CI, 0.0.08–0.0.19, *p* < 0.001) and young adult (OR = 0.47, 95% CI, 0.35–0.62, *p* < 0.001) decreased the risk of relinquishing an animal compared to adult dogs, while being a senior increased the risk (OR = 1.89, 95% CI, 1.49–2.39, *p* < 0.001). The risk for relinquishment was decreased in small population centers (OR = 0.65, 95% CI, 0.51–0.83, *p* < 0.001) compared to large population centers, and smaller dogs (OR = 1.82, 95% CI, 1.49–2.22, *p* < 0.001) were more likely to be relinquished for the reason of “guardian health” compared to large dogs (Figure 9).

## Discussion

As we predicted, various human and animal factors increased the risk of relinquishing dogs for particular reasons. Over the last decade, while the total intake of dogs to BC SPCA shelters has declined over time, the relinquishment of dogs made up 31–35% of total intake and thus remains a consistent proportion of shelter intake. These BC-specific proportions are similar to estimates of the Canadian national average reported by Humane Canada surveys (16) and to that reported in the USA (1, 37). As reflected in previous studies [e.g., Jensen et al. (7)], the reasons that guardians relinquish their dogs to shelters in BC are predominately a result of the guardian's circumstances, not because the animal is unwanted or for reasons relating to the animal (7, 8). Shelters, such as BC SPCA shelters, can support human and animal welfare by serving as a safety net for guardians who are best served by maintaining the option to relinquish, but may also wish to offer support to the likely many guardians who would prefer to find an alternative to relinquishing to an animal shelter. As the proportion of dogs relinquished by guardians was relatively consistent over time, exploring regional and characteristic-specific reasons for relinquishment provides insight into specific intervention strategies to prevent intake.

The primary reason reported by guardians for the relinquishment of dogs to the BC SPCA between 2008 and 2019 was the guardian having too many animals, with an increase in this reason for relinquishment over time. Puppies were at an exceptionally greater risk for relinquishment for this reason compared to adult and senior dogs, and smaller dogs were at a greater risk of relinquishment than larger dogs. Dogs in small and medium population centers compared to large population centers had a greater risk of relinquishment for this reason, and suspected purebred dogs were less likely to be relinquished compared to suspected mixed breeds. While adopter demand remains likely to meet the intake of dogs to shelters at a provincial level, these results demonstrate that this primary reason for the relinquishment of having too many animals remains a part of the landscape across BC.

While research has not been conducted on the attitudes toward sterilization in communities in BC, little difference was found in attitudes toward sterilization across rural and urban areas in the USA (38). While relinquished dogs in BC showed higher overall proportions of sterilization on intake than in Mexico (39), a similar difference was observed in BC data, with dogs entering the shelter from large population centers being more likely to be previously sterilized than dogs from small and medium population centers. Increased odds of relinquishment for the reason of too many animals in small population centers, many of which are in remote areas of BC, may suggest an issue with access to care for sterilization procedures. In BC, shelters and other non-profits can offer services essential to addressing this problem, such as targeted low-cost or subsidized care.

Housing issues as a reason for relinquishment was found to be particularly pronounced in large population centers, which may present promising areas for further research and intervention. While evidence of housing interventions is scarce, occasional creative solutions have been described. For example, The San Francisco SPCA spearheaded a trial in an urban rental building offering to financially reimburse landlords for any damages incurred due to allowing pets in their building and ultimately were asked to pay no money in damages (40). Weiss et al. (5) found that in guardians relinquishing their companion animals, access to temporary boarding or pet-friendly housing may have helped them retain their pets. In some cases, preventing guardian relinquishment may be as simple as providing a guardian with a pet deposit (23). Unique solutions such as these and encouraging local governments to increase the accessibility of inclusive pet-friendly housing (devoid of restrictions on size, number and breed) by creating less pet-restrictive laws provincially would likely help to decrease the continually large number of guardian relinquishments of all dogs, especially dogs in large population regions in BC.

Personal issues resulting in relinquishment in BC are primarily due to an inability to provide animal care, lack of time, or too much responsibility. This aligns with current research showing that time pressures of dog care due to daily needs and frequent walks are a common reason for relinquishment (7). Puppies, TR, TM or UU Asilomar Accords category, and female dogs are less likely to be relinquished for guardian personal issues, demonstrating that a variety of dogs of varying characteristics are consistently relinquished for personal issues across BC. In large population centers, dogs were more likely to be relinquished for guardian personal issues. It is possible that characteristics of large population centers, where people and dogs generally have more limited individual space and outdoor access, may contribute to challenges providing necessary animal care, resulting in relinquishment.

The most commonly reported animal-related reason for the relinquishment of dogs is behavior, with objectionable activities such as hyperactivity, barking, destruction, and escaping making up the majority of reasons within this category. Puppies and senior dogs were less likely to be relinquished for behavior issues, likely indicating that problem behaviors were either less likely to be displayed or more likely to be tolerated. This is consistent with a previous study demonstrating that dogs over 6 months of age were more likely to be relinquished for behavior issues (20). Smaller dogs were also less likely to be relinquished than larger dogs. Cannas et al. (41) found that smaller dogs tended to be referred to a behavior clinic for anxious behaviors, while larger dogs were more likely to be referred to a behavior clinic for aggressive behaviors. Behavioral differences may be observed between dog sizes. Alternatively, certain behaviors may be more likely to be perceived as a problem resulting in the relinquishment of larger dogs, such as hyperactivity, destruction, and aggression, all of which are commonly reported in BC relinquishments.

As with personal issues (which were most commonly related to the dog being too much responsibility or time commitment), it was expected that behavior problems may be more likely to arise or present a more prominent problem for the guardian in large population centers. Julien et al. (42) found that people living in urban environments in Southern Ontario had higher odds of being bitten by a dog than people living in rural settings, and suggest this may be due to limited green space, likely smaller homes, and more time indoors, leading to more negative behaviors. In a study surveying dog behavior during COVID-19 pandemic lockdowns, guardians who experienced lockdown or quarantine and did not have a garden were more likely to experience dog care and behavior difficulties (43). However, while this pattern was observed for relinquishment due to personal issues, population center size did not predict relinquishment for behavioral reasons.

There is limited research investigating intervention strategies to prevent behavioral relinquishment of dogs (44). As approximately only 15% of dog guardians seek professional help for behavioral issues, shelter intervention could connect dog guardians to behavioral resources and community programs, as well as trainers or pet care professionals that may be able to assist with behavior or care problems (23). Powdrill-Wells et al. (45) found that 24.4% of guardians accepted advice among guardians relinquishing for behavior and were more likely to accept advice for general behavior issues such as jumping than aggression between dogs in the home. When escaping is the issue, solutions such as helping to fix a fence could offer the potential for a dog to remain at home. For behavioral issues such as house soiling, for example, intervention opportunities may be possible as these issues are likely solvable if caught early (23). Targeted intervention in the form of, for example, accessible dog-walking services may offer a promising area of increasing dog retention. However, further research into the specific needs of guardians in these circumstances would be beneficial for informing specific intervention needs.

Dogs in the Asilomar Accords categories of TR, TM or UU were especially at risk for relinquishment for reasons relating to cost compared to H, demonstrating the cost of veterinary care for health issues being a clear burden for many people. The least commonly reported reason for relinquishment was animal health, likely indicating that when an animal has a health issue resulting in relinquishment, the reason for relinquishment is often related to the cost of care, not due to the issue itself, as only 148 dogs were relinquished to the BC SPCA between 2008 and 2019 with the guardians citing the health issue alone, not the cost of caring for the health issue. While a direct link between veterinary services and animal relinquishment has not been explored, spay-neuter efforts have been demonstrated to be useful in spay-neuter saturation among animal populations in New York City (31). Given that veterinary costs make up the majority of reasons for cost-related relinquishment of dogs in BC, expansion of ongoing efforts to provide low-cost and free veterinary services in the community may provide further opportunities to keep dogs with their guardians.

Senior dogs, dogs in large population centers, and small dogs were more likely to be relinquished for the reason of guardian health. As this reason is primarily guardian-related not animal-related, these observed trends in relinquishment are likely related to the preferences of the guardians expected to relinquish for this reason (including size of dog, and region of residence). Detailed demographic information of the relinquishing guardian was unknown. However, the population relinquishing for human health may be more likely to include elderly guardians. Therefore, they may also be more likely to have a senior dog, live in a larger population center or prefer smaller dogs. Intervention strategies when guardians are experiencing health issues may be essential when suitable due to the emotional and health benefits of having a companion animal, especially for elderly populations (46). While some relinquishments for guardian health are unavoidable (e.g., death), other intervention strategies such as temporary boarding during illness may be beneficial in keeping dogs and guardians together.

Finally, a common potential concern with relinquishment is that giving an animal as a gift may lead the animal to end up in a shelter as an “unwanted gift” or that dogs are relinquished for being unwanted or no longer wanted. However, studies have repeatedly demonstrated that animals given as gifts do not seem to be at increased risk of relinquishment to shelters, and relinquishment for the reason of being unwanted is scarcely reported (13, 19, 47). As only 4% of dogs were relinquished to the BC SPCA for being no longer wanted, this further shows that guardians predominately wish to keep their dog, but need to resort to relinquishment due to their circumstances.

The data collected in this study included only guardian-volunteered information provided at the time of relinquishment; hence, data collection methods may have varied somewhat across shelters, staff members, and over time. Furthermore, as the information is guardian-provided, some reported reasons may not accurately reflect the true motivation, as some relinquishment reasons (e.g., moving) may be more socially acceptable to report than others (48). Therefore, some reasons may have been over-reported. Relinquishment for the reason of being a community dog (defined as a dog cared for by a community) may have been recorded as stray upon intake. Therefore, this category is likely underrepresented in these data, hence making dynamics related to community dog relinquishment difficult to assess. Additionally, there may have been cases where many factors influenced the decision to relinquish an animal, so the primary reasons reported by guardians (as captured in the data collection) may not fully reflect the actual situation. Finally, factors known to impact relinquishment reason, such as human vulnerabilities including the ethnocultural composition of the originating neighborhood, were not included in the analysis (25), and likely contributed to reasons for relinquishment within each area beyond population.

Despite these limitations, the patterns illustrated from these data show consistent trends in the reasons of guardian relinquishment to BC SPCA shelters. While across BC, relinquishment of dogs for all reasons occurs, particularly promising region-specific intervention opportunities are present. These include efforts to prevent too many animals or overpopulation in small population centers and improve pet-inclusive housing options, with a specific focus on improving housing options for large population centers. Furthermore, daily care support for dog guardians in large population centers demonstrates a promising area for potential intervention. Finally, providing subsidized, low-cost, and accessible veterinary services likely benefits dog guardian retention across BC, regardless of region.

## Data Availability Statement

The original contributions presented in the study are included in the article/Supplementary Material, further inquiries can be directed to the corresponding authors.

## Ethics Statement

As this project included analysis of data excluding human identifiers, this project was deemed exempt from the University of British Columbia Behavioral Research Ethics Board review.

## Author Contributions

BE, EG, and AP contributed to the study design. BE acquired and organized the database and wrote the first draft of the manuscript. BE and AP performed the statistical analysis. All authors contributed to the manuscript, further revisions, and approved the final version of the manuscript for submission.

## Funding

This project was supported, in part, by the Natural Sciences and Engineering Research Council of Canada and the British Society for the Prevention of Cruelty to Animals (Industrial Research Chair in Animal Welfare #554745-19).

## Conflict of Interest

The authors declare that the research was conducted in the absence of any commercial or financial relationships that could be construed as a potential conflict of interest.

## Publisher's Note

All claims expressed in this article are solely those of the authors and do not necessarily represent those of their affiliated organizations, or those of the publisher, the editors and the reviewers. Any product that may be evaluated in this article, or claim that may be made by its manufacturer, is not guaranteed or endorsed by the publisher.

## Supplementary Material

Supplementary Data Sheet 1 Eagan et al. Code. Github link for access to R code used to analyze the reasons for guardian-relinquishment of dogs to shelters: animal and regional predictors in British Columbia, Canada.

Supplementary Data Sheet 2 Eagan et al. Data File. BC SPCA guardian relinquishment of dogs 2008-2019.

Supplementary Data Sheet 3 Eagan et al. Data File. Model sample sizes.
