# Supplementary material for: Reasons for Guardian-Relinquishment of Dogs to Shelters: Animal and Regional Predictors in British Columbia, Canada
Source: Front Vet Sci. 2022 Apr 14;9:857634. doi: 10.3389/fvets.2022.857634 (PMC9050194; doi:10.3389/fvets.2022.857634)
Supplement: Supplementary Data Sheet 1 — Eagan et al. Code. Github link for access to R code used to analyze the reasons for guardian-relinquishment of dogs to shelters: animal and regional predictors in British Columbia, Canada. [file Data_Sheet_1.PDF]

SUPPLEMENTARY DATA SHEET 1

Access to R code for "Reasons for Guardian-Relinquishment of Dogs to Shelters: Animal and Regional Predictors in British Columbia, Canada" available at: https://github.com/baileyhe/dogrelinquishment.
